# Commentary: Hemophagocytic lymphohistiocytosis induced by immune checkpoint inhibitors: observations and proposed clinical management

**DOI:** 10.3389/fimmu.2026.2009423

**Published:** 2026-10-26

**Authors:** Gauthier Coussement, Maxime Ilzkovitz, Loïc Baudelet, Anne-Pascale Meert, Louis Wolff

**Affiliations:** 1 Department of Internal Medicine, Institut Jules Bordet, Hôpital Universitaire de Bruxelles (H.U.B.), Université Libre de Bruxelles (ULB), Brussels, Belgium; 2 Belgian Multidisciplinary Immunotoxicity Board (BITOX), Brussels, Belgium; 3 Immunotoxicity Management Competence Centre, Université libre de Bruxelles (ULB), Brussels, Belgium

**Keywords:** anakinra, hemophagocytic lymphohistiocytosis, HLH, IL-6, immune checkpoint inhibitor, irAEs

We read with great interest the perspective by Facchini et al. proposing a diagnostic and therapeutic algorithm for immune checkpoint inhibitor (ICI)-associated hemophagocytic lymphohistiocytosis (HLH). Given the rarity of this immune-related adverse event (irAE), the authors should be commended for attempting to provide a pragmatic framework for clinicians.

However, we believe that several aspects of the proposed therapeutic algorithm warrant further discussion. The authors acknowledge the limited evidence for second-line treatment and allow clinical discretion when IL-6 testing is unavailable. First, the rationale for selecting IL-6 blockade over IL-1 blockade according to a serum IL-6 threshold (>20 pg/mL) deserves greater scrutiny. To our knowledge, no study in HLH, including ICI-associated HLH, has established a validated IL-6 cutoff capable of predicting response to tocilizumab or identifying patients in whom IL-6 is the dominant inflammatory driver. Current HLH diagnostic criteria (HLH-2004 (1), HLH-2024 (2), HScore (3)) do not incorporate IL-6 measurements, and international recommendations similarly do not advocate cytokine-guided therapeutic decisions (4, 5). Furthermore, circulating IL-6 concentrations are characterized by substantial biological variability, including circadian fluctuations, rapid changes during acute inflammation, and assay-dependent variability (6). IL-6 levels have also been shown to be more elevated in sepsis than in HLH, potentially limiting their usefulness as a decision-making biomarker when sepsis remains under consideration (7, 8). No definitive IL-6 cutoff has been validated with different values used for different diagnoses and values varying across published series and laboratories ranging from 4.2 ± 2.1 to 203 pg/mL (7, 8). Moreover, various degrees of IL-6 elevation have been described in an array of different neoplasms with associations between its elevation and prognosis in different diseases (9, 10). Without a baseline value obtained at diagnosis, an elevated IL-6 level may therefore be difficult to interpret. Serum concentrations may also inadequately reflect tissue cytokine activity, which is likely to be more relevant in hyperinflammatory syndromes. Consequently, using a single IL-6 value to determine whether a patient should receive tocilizumab, an IL-6R antagonist, rather than another immunomodulatory agent appears insufficiently supported by current evidence.

Given the common pathways activated in hyperinflammatory states such as cytokine release syndrome (CRS), HLH, and sepsis, the simultaneous use of multiple biomarkers to support diagnosis and guide treatment may prove valuable (7). In a small cohort, a ferritin threshold >23,111 ng/mL achieved 100% accuracy in distinguishing ICI-associated HLH from sepsis and CRS (7, 11). However, this exploratory cutoff requires external validation.

Second, the proposed algorithm underrepresents the available evidence supporting IL-1 inhibition. Although high-quality comparative data are lacking, anakinra has accumulated increasing observational evidence across secondary HLH and macrophage activation syndrome (MAS), with a favorable safety profile and the advantage of preserving host antimicrobial immunity better than broad cytotoxic approaches (12–15). Indeed, several contemporary reviews and expert recommendations consider anakinra among the preferred second-line agents for secondary HLH, particularly in infection-associated or rheumatologic settings, even though evidence specifically addressing ICI-associated HLH remains limited (4, 12, 13, 16–18). The 2022 EULAR/ACR points to consider include high-dose glucocorticoids, anakinra, and/or IVIG as options for initial empiric immunomodulatory treatment of suspected HLH/MAS (4). Furthermore, its short half-life offers the possibility for a more adaptive approach with repeated doses, and both subcutaneous and IV administrations have been described (4, 16–18). Presenting IL-1 blockade as merely one option among several, while assigning a central role to IL-6-guided therapy, may therefore overstate the current evidence supporting the use of tocilizumab.

Finally, ICI-associated HLH may represent a distinct HLH-like hyperinflammatory phenotype. However, this hypothesis requires further validation. In clinical practice, some cases occur in association with concurrent severe irAEs such as immune-mediated colitis, hepatitis, pneumonitis, or myocarditis, while simultaneously fulfilling HLH diagnostic criteria through extreme hyperferritinemia, cytopenias, fever, and sometimes elevated soluble IL-2 receptor. Reported mortality was 15% in ICI-associated HLH versus 48% in non-ICI-associated HLH cohorts (19, 20). However, this indirect comparison may be influenced by differences in patient populations, HLH triggers, diagnostic criteria, and treatments. This overlap raises the possibility that, in at least a subset of patients, the HLH-like manifestations in ICI-treated patients represent one manifestation of a broader systemic immune activation syndrome induced by checkpoint inhibition, rather than classical secondary HLH driven by the same immunopathological mechanisms as malignancy- or infection-associated HLH. Future studies should explore the timing of ICI exposure, concurrent irAEs, cytokine profiles, clinical course, and treatment response to better characterize this potential phenotype.

A multiple-biomarker approach might represent an interesting strategy; however, its application might be limited by the rapid changes in circulating cytokines as well as the availability and the time required to obtain results in most centers. More readily available and less labile markers such as ferritin might be a more solid approach pending more robust data.

Overall, we applaud the authors for addressing a clinically important and challenging toxicity. However, given the current absence of validated biomarkers and prospective therapeutic studies, we believe that cytokine-directed treatment algorithms should avoid relying on unvalidated serum IL-6 thresholds and should more explicitly acknowledge both the growing role of IL-1 blockade and the emerging concept that ICI-associated HLH may represent an HLH-like manifestation within the spectrum of severe immune-related adverse events. These uncertainties further highlight the need for prospective multicenter registries incorporating standardized cytokine profiling, tissue biomarkers, and treatment outcomes to better define optimal management strategies.
